# Neural signatures of word learning during adult-child interactions

**DOI:** 10.1162/imag_a_00407

**Published:** 2025-01-02

**Authors:** Sara Mosteller, Sobanawartiny Wijeakumar, Sam Wass

**Affiliations:** School of Psychology, University of East Anglia, Norwich, United Kingdom; School of Psychology, University of Nottingham, Nottingham, United Kingdom; School of Psychology, University of East London, London, United Kingdom

**Keywords:** language, development, word learning, functional near infrared spectroscopy, hyperscanning

## Abstract

Early language acquisition relies on the successful communication of specific word meanings when speaking or encoding labels. Here, we tested for evidence of this communication within the parallel functional neural activation that occurred for adults and children as unfamiliar objects were labelled by the caregiver. Measuring event-related hemodynamic responses with functional near-infrared spectroscopy (fNIRS) simultaneously recorded from both individuals, we compared cortical responses that were time-locked to spontaneous instances of object labelling during openly structured interactions. Most notably, children’s responses differed between words learned and not learned in the right posterior temporal cortex, with relatively greater positive activation when children were not learning. The findings bear relevance to investigations of coordination between speakers and listeners during word-learning interactions

## 1 Introduction

Early language acquisition occurs through the conveyance of specific word meanings between speakers and listeners during dialogue. As speakers refer to objects in conversation, children must generally draw from the situational context, their language experience and social skills to interpret these references and ultimately assimilate unfamiliar words into their rapidly growing vocabulary. Past work has indicated that this ability involves resolving multiple types of demands rather than simply reasoning about language syntax. For example, relevant work across differing language tasks uncovered functional neural activation not only in language-predominate networks but also in functionally heterogeneous networks that reflected a broader range of underlying processes (Bašnáková et al., 2014;Egorova et al., 2016;Feng et al., 2017;Lipkin et al., 2022;Redcay et al., 2016;Van Petten & Luka, 2006). The impacts of interactive experience on language also seem to be present in various types of observations from early in life, as greater activation in social processing regions like the temporoparietal junction (TPJ) were observed when children*believed*they were listening to a live speaker as opposed to when they knew the same speech was pre-recorded (Rice et al., 2016) while amounts of turn taking in conversation at home were shown to be predictive of more gradual myelination of tracts within the language network (Romeo, Segaran, et al., 2018). Yet, research to date has not adequately captured spatially precise neural responses to single, unfamiliar words occurring in-the-moment as children form associations between these words and their referents during the interactive dynamics of dialogue. Instead, in prior work, word learning and sentence processing more generally were predominately observed from presenting participants with speech recordings.

Studies that were more linearly distributed across ages examined neural responses to single words and sentences from infancy through adulthood. In such tasks, shifts were observed in the event-related amplitudes of EEG recordings from infants when hearing isolated words and following even a few presentations. This suggested the potential presence of some early and rapidly occurring, if weakly retained, associations made between new words and corresponding objects (Friedrich & Friederici, 2008,^2011^,^2017^). When 6-month-old infants heard nine presentations of new labels paired with novel objects, their event-related EEG data showed widespread and left-biased frontal decreases in the relative amplitude of responses post-learning that were temporally locked to hearing the noun, while their fNIRS data showed bilateral frontal and temporal learning effects. The observed effects occurred across words that were both phonetically likely and unlikely (Obrig et al., 2017). From here, MRI studies have shown that by 3 years, sentence processing activates the left-lateralised language network much like in adulthood (Vissiennon et al., 2017). Most work from this point has scanned children no younger than 4 to 5 years of age, generally finding consistencies between adults and children in language processing (Berl et al., 2014;Ekerdt et al., 2020;Nora et al., 2017;Szaflarski et al., 2012;Wang et al., 2022).

Additionally, while behavioural research reveals situationally relevant information sharing between teachers and learners during successful learning (Chen et al., 2021;Hawkins et al., 2020), related neuroimaging work has largely focused on the perspective of the learner, or the receiver of information, rather than that of the sender. Yet, some work suggests that relationships in brain activity between a speaker and listener may be predictive of successful communication (Liu et al., 2019;Pérez et al., 2019;Stephens et al., 2010), a hypothesis that has further been supported in a few developmental studies to date (Piazza et al., 2021;Zhai et al., 2023). At present, it remains an open question whether neural responses that evidence teaching new words during object naming can be reliably observed in relation to children’s learning outcomes.

This study aimed to address existing methodological gaps in examining neural responses to the naming of new objects between 2 and 5 years of age and during openly structured interactions through a fully naturalistic neuroimaging session. Using a wearable neuroimaging technology (fNIRS) that is relatively robust to movement (Herold et al., 2017;Yücel et al., 2021), we have captured a developmental window of language acquisition between 2 and 5 years of age, within dyadic interactions. Caregivers taught their 32- and 54-month-old children the names of eight unfamiliar objects during openly structured play while fNIRS signals were recorded from each. Participants were then tested on their memory for the learned object-label pairings using an age-appropriate test in which children selected objects from pairs containing a target and distractor object (Axelsson et al., 2016;Samuelson et al., 2011), while adults were asked to name the objects learned throughout the study at the very end of the session. Later, as part of the analysis, the child’s and caregiver’s event-related hemodynamic responses following instances in which the caregiver named objects during these interactions were compared between the child’s learned words and their words not learned, as indicated by the child’s choices in the referent selection test. As such, the aim was to keep the task and measures of learning as close as possible to those that had been established in past studies of word learning and to the event-related analysis methods used in related cognitive neuroscience studies. However, these established methods were applied to word learning during naturalistic interactions.

The first question asked was: how do the neural correlates of word learning from hearing objects labelled develop for children up to 5 years of age, given that most past neuroimaging work that had assessed neural correlates of early semantic processing with spatial precision had started at 4-to-5 years of age (Berl et al., 2014;Ekerdt et al., 2020;Nora et al., 2017;Szaflarski et al., 2012;Wang et al., 2022, but seeRedcay et al., 2008;Vissiennon et al., 2017, for studies with younger children). Both age groups chosen in this study were expected to be skilled enough at word learning to complete the task involved. To assess how similarly and differently the process of word learning unfolded between these ages (as compared with instances of naming that did not result in learning), we measured event-related hemodynamic responses as children were taught new word-object pairings, then assessed children’s memory for each pairing in a referent selection task. The event-related averages to naming of unfamiliar objects during the interactions would then be contrasted between those of words learned by the child versus those of words not learned.

Based on the limited past work available, we predicted that differences in activation for children between the naming of learned words versus of words not learned would be observed within the language processing network, which commonly peaks in the left inferior frontal gyrus (IFG) and the left temporal cortex (Lipkin et al., 2022;López-Barroso et al., 2013;Romeo, Leonard, et al., 2018;Romeo, Segaran, et al., 2018) or within the same areas in the right hemisphere (Berl et al., 2014;Nora et al., 2017;Redcay et al., 2008). However, coverage with the NIRS cap was most certain over the inferior frontal gyrus, given the proximity of areas of the temporal cortex involved in speech processing to the ears. Conducting our analysis over the full recorded area that was inclusive of cortical regions involved in attention, working memory and social processing, enabled us to examine a richer range of responses to naming events. Therefore, we left open the possibility that this full analysis would uncover differences in other regions associated with different kinds of functions (not explicitly language-related). Though we planned to examine age as a factor with the 32-month-old children being more distant from the ages tested in most similar MRI studies, we made no hypotheses about similarities or differences between the age groups.

A second gap in the literature was the need to measure these correlates during openly structured interactions, rather than via more constrained, computer-based word learning tasks. Because the literature was even more limited, no predictions were made regarding the question of how these interactions might shape the differences observed between event-related hemodynamic responses to words learned or not learned. Finally, a third question was: are there neural signatures of successful teaching that differentiate adults’ neural responses during object naming from instances of unsuccessful teaching? Such a question takes a step toward addressing the gap described previously, of investigating how and to what extent a speaker’s neural responses to their naming of objects during dialogue in which they are teaching their child can be related to the listener’s learning. To investigate this question, we planned to conduct a parallel analysis of the caregivers’ hemodynamic responses to the naming of words their child learned, versus hemodynamic responses to the naming of words their child did not learn. No prior predictions were made about this analysis because before conducting this study, the question had remained completely un-investigated in prior work.

## 2 Methods

### 2.1 Participants

Participants were nineteen 32-month-olds (M=32.5months,SD=52days), 18 54-month-olds (M=53.7months,SD=58days), 19 male, and their caregiver/parent, all female (for a total of 74 participants), all hearing and speaking English at least 50% of the time, recruited from a city and surrounding areas in Norfolk, UK. Data were excluded from an additional 24 dyads because the child did not want to wear the NIRS cap to participate, and from 7 dyads because of technical errors that occurred during testing.

### 2.2 Ethics statement

The research was approved by an Ethics Committee in the School of Psychology at the University of East Anglia. Participants were informed about the study ahead of time, including data confidentiality, and caregivers provided verbal and written consent for themselves and their child to participate. In addition, children could opt out by expressing at any time that they did not wish to participate.

### 2.3 Testing materials

In a single session, children completed a British Picture Vocabulary Scale version 3 (BPVS-3) assessment of general vocabulary (Dunn & Dunn, 2009), and then the dyads engaged in a series of brief learning interactions, followed by an assessment of learning from an experimenter. During the interactions, caregivers and children were seated across from each other at a 40” by 24” table, as shown inFigure 1. The session was videotaped from an overhead camera that was mounted to the ceiling and a camera placed near the table that also recorded audio. Photos of objects and their labels were presented on a felt board positioned within the adult’s view, but out of sight of the child.

**Fig. 1.**
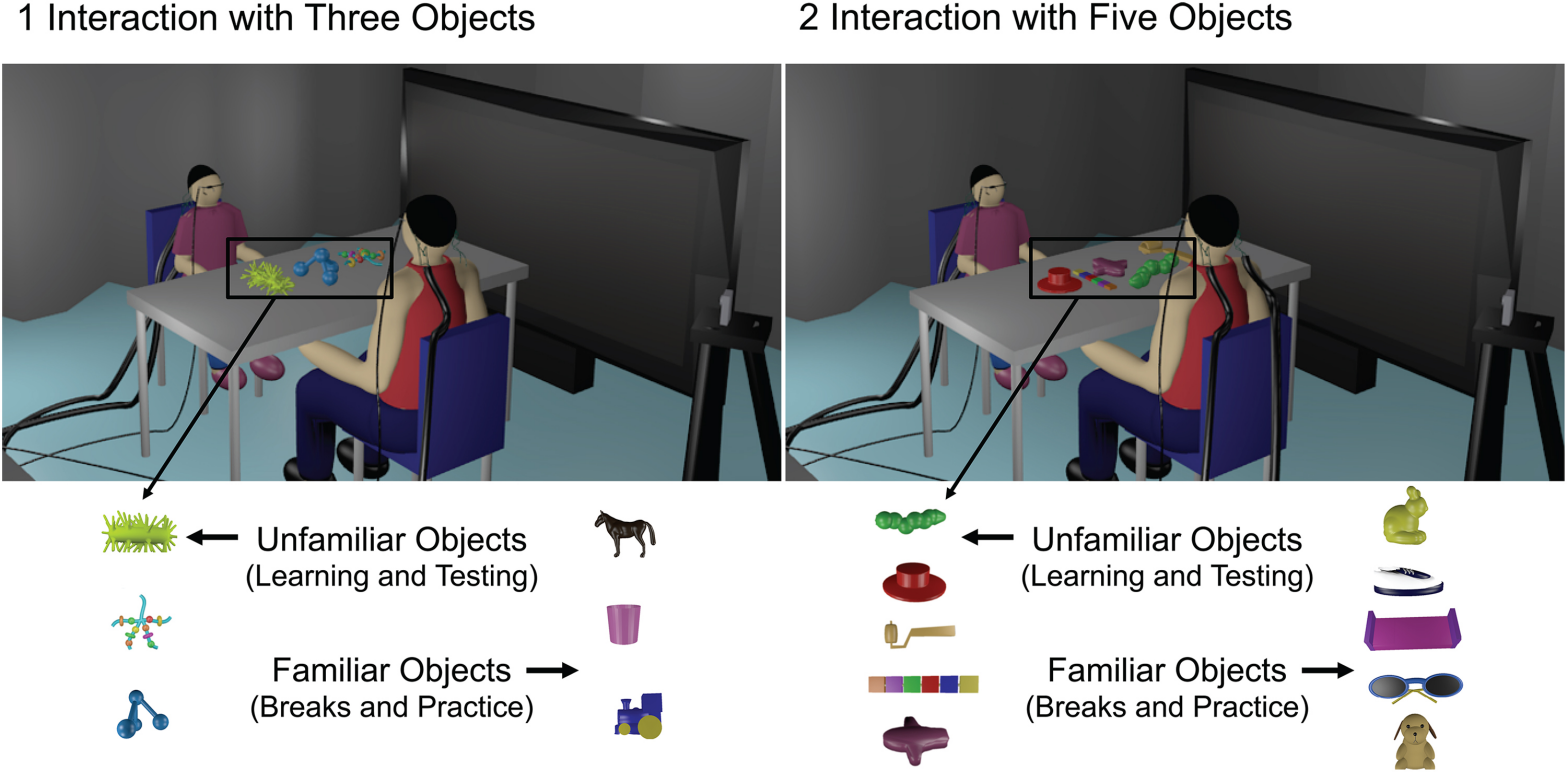
An Illustration of the Experimental Setup and Objects Used. Note. The images show (1) an interaction with three unfamiliar objects and (2) an interaction with five unfamiliar objects. Below, the three-object set of unfamiliar and corresponding familiar objects (left), and the five-object sets (right). All dyads interacted with, and were tested on the labels for, both sets of unfamiliar objects during the session, as shown below. As shown in the model, fNIRS recordings were acquired from both participants during the interactions with the unfamiliar objects, while the session was videotaped so as to later mark the naming events and the child’s selections during the learning assessment.

Eight unfamiliar objects were placed on the table between the adult and the child. These consisted of a three-object set and a five-object set of toys. The smaller three-object set was made up of a yellow squishy toy, a large decorative key ring, and a blue plastic structure. The larger five-object set was made up of a green wooden caterpillar, a miniature paint roller painted gold, a red mesh structure, a set of brightly coloured blocks, and a small felt puppet. The differently sized sets were chosen in order to include a task that was likely to be easy enough that 32-month-old children learned a few words on average (Axelsson et al., 2016) but that was also likely to be challenging enough that most 54-month-old children would not learn all of the words.

Each unfamiliar object was matched to an unfamiliar label. The unfamiliar labels were chosen from the Novel Object and Unusual Word (NOUN) database (Horst & Hout, 2016). The pairing between unfamiliar objects and unfamiliar labels was counterbalanced across participants. In addition, familiar toys were also used as a break in between learning the labels of the unfamiliar object set. These trials were also used to assess that the child was on-task during the later learning assessment. These familiar toys consisted of a three-object set of a toy horse, shoe, and cup and a five-object set of toy train, dog, rabbit, bed, and sunglasses.

### 2.4 fNIRS data acquisition

A TechEN continuous wave (version 7) system was used to collect brain function data (wavelengths of 690 nm and 830 nm and a sampling frequency of 25 Hz). Light was transmitted from the system to the cap worn by the participant through 16 fibreoptic cables and measured by 32 detectors, resulting in 20 identical channels each for the adult and child. The field of view from this geometry covered the superior frontal cortex, inferior frontal gyrus, posterior temporal cortex, and parietal cortex. An additional overcap was placed over the NIRS cap.

### 2.5 Procedure

One experimenter explained the task to the adult, while a second experimenter completed the BPVS-3 with the child. The adult was shown where they could reference the pictures of the objects and labels and encouraged to teach their child the labels while interacting with them as naturally as possible. No further instructions were given to the adults about how to teach their child the labels; therefore, there was no prescribed way for them to teach their child. Caregivers indicated whether their child had used any label for any of the unfamiliar objects, and it was noted if they did. This occurred only rarely (<5% of cases). Both participants were then fitted with a NIRS cap. A flash from the computer monitor was used to synchronize the video recordings with the NIRS recordings and to mark the pre-specified beginning and the end of each interaction.

First, adults were asked to interact with their child to teach them labels for the first set of toys. There were two segments of these interactions, each lasting 2.25 min (45 sec per object * 3 objects). The naming events during these openly structured interactions were used as trials in the later analysis. In between the interactions, the participants were given the familiar objects to interact with for 1.5 min. In total, the participants had an estimated 10 min between learning and a test of comprehension, via a break where an experimenter asked the child to select and name the familiar objects for an additional 1.5 min, and needed changes to the setup. The experimenters then began a test of comprehension, for which the child was presented with pairs of familiar objects alternating with pairs of unfamiliar objects and asked to select one of the objects during each presentation, such that each object was asked for twice and appeared alongside the object asked for twice. For example, the child may be presented with the horse and cup in the first trial and asked to retrieve the horse. Noting that the child was following instructions, the experimenter would then present them with two unfamiliar objects side by side and ask for the “modi”, the taught label for one of the objects. The test consisted of 12 trials in total; during 6 of these, the child was asked to retrieve a familiar object from a set of two familiar objects and during 6 of these, an unfamiliar object from a set of two unfamiliar objects. Next, the setup was returned to a face-to-face interaction between the dyad and the caregivers were again asked to interact with their child to teach them labels for the second set of toys. There were two segments of these interactions, each of duration 3.75 min (45 sec per object * 5 objects), and again, the dyads interacted with the familiar objects in between for 1.5 min. Then, the experimenters moved the adult to face away so as not to interfere with the child’s object selections, asked the children to name the familiar objects during a brief interaction (1.5 min), and finally conducted a test of comprehension. The test consisted of 20 trials in total; during 10 of these, the child was asked to retrieve a familiar object from a set of two familiar objects and during the remaining 10 trials, an unfamiliar object from a set of two unfamiliar objects.

After the interactions, scalp landmarks and optode locations were digitised from both the adult and child. Lastly, after the child had left the testing room, the adult was asked to recall the names of the objects to assess their learning. Each object was held up for the adult to name in sequence, and in each case, they were given around 12 sec to answer.

### 2.6 Coding of the naming events during the interactions and learning assessments

Whenever the objects were named during the interactions, these events were selected within a waveform of the speech recording. The unfamiliar object label was selected as a single-word portion, from its onset to its offset. The onsets of these events, that marked the first syllable of each object label as it was spoken, were later used as individual trials in the analysis, divided into two categories based on whether the child had learned the label (words learned, words not learned).

Children’s learning was determined based on performance during the assessment. During this test, if the child selected the correct object on both trials for a given label, that label was marked as learned. The adults’ learning was only used as a secondary measure at the beginning of the analysis, to determine the extent to which the adults and children learned the same words or learned words at a similar rate. We scored this based on their ability to recall object labels during a production test at the end of the experiment, and all reproductions marked as at least moderately, rather than only vaguely, accurate were counted as learned.

Data from 22% of participants in the study were double-coded. The onsets and offsets of naming events were marked within a 250 ms window of accuracy with a 98% agreement between independent coders. Likewise, for the comprehension test, coders agreed on which object the child selected, with a high overall inter-coder reliability of 99%. Finally, coders agreed on the adults’ accuracy in naming each object within one level of a five-point Likert scale with an overall reliability of 99%.

### 2.7 fNIRS pre-processing

The onsets of the adults’ naming events from all of the interactions with unfamiliar objects were inserted into the fNIRS recordings collected from both the adult and child. These events were assigned within a binary event coding scheme based on whether the child had correctly selected the referent for the word spoken or did not learn the word. The mean and distribution of the median intervals between adjacent naming events by dyad and learning are shown inFigure 2—these were the spacing of events for the general linear model in the study. Overall, adults’ naming across all of the unfamiliar objects within the 6 min of total interaction time was quite frequent,M=101total naming events,SD=38,range=45−183total naming events. fNIRS data were pre-processed in MATLAB using HomER2 software (Huppert et al., 2009). Channels with raw intensity values less than 70 dB or greater than 140 dB were pruned. Intensity values were then converted into optical density (OD) units. Next, motion artefacts were identified and corrected using a targeted principal component analysis with the following parameters:tMotion=1,tMask=1,STDEVthresh=50,AMPthresh=0.4,nSV=0.97,maxIter=5(Yücel et al., 2014). The data were bandpass filtered to a range of 0.5-0.016 Hz. Any markers overlapping with uncorrected motion artefacts were removed. After pre-processing, around 80% of the children’s and around 70% of the adults’ data met these criteria and were retained for the analysis. The relationship of the masks to the channel locations in the scalp is further illustrated inFigure S2.

**Fig. 2.**
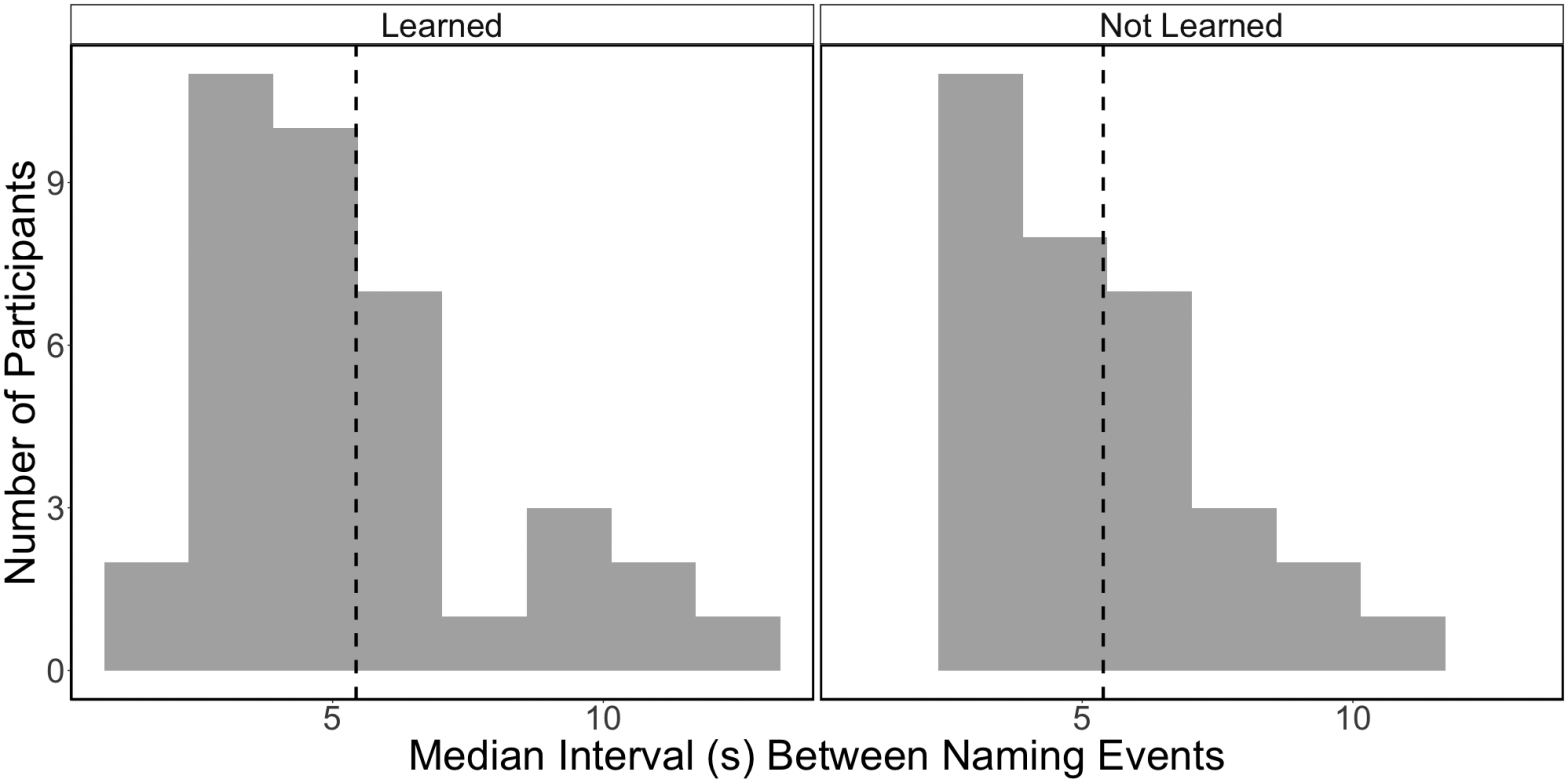
Intervals Between Naming Events. Note. The distribution of time intervals between adjacent naming events. Histograms show distributions of the median inter-trial interval, or distance between naming events for each participant, by learning outcome. The mean of these values is shown by a dashed line for each group.

### 2.8 Image reconstruction techniques and general linear model (GLM)

To determine the cap geometry, searches were made in the PsychINFO and PubMED databases (with checks run in Google Scholar), using terms such as “rapid word object mapping”, “novel word learning”, and “child word learning”, as well as “social interaction” and “social language development”. We also considered a meta-analysis of the neural correlates of visual working memory (Wijeakumar et al., 2015,Table 2). From here, we used the process described inWijeakumar et al. (2015)to overlay the 46 distinctive regions uncovered in these studies with potential cap geometries for a 30-month-old child and an adult so as to optimise coverage of relevant regions. This was done using the AtlasViewerGUI function in the HomER2 software. Previous work has shown that different source-detector separations are necessary for infants and adults due to anatomical differences in skull thickness (Lloyd-Fox et al., 2010). To map this scaling across age, standard reference points on the caps were then scaled to the different cap sizes. Thus, the probe geometry, anchored to these points, was scaled down to differing cap sizes. We estimate that for children, source-detector distances ranged from 2.4 cm–2.6 cm, while for adults, they ranged between 2.7 cm and 3.0 cm.

During the session, digitised scalp landmarks and optode locations from participants were transformed to correct measurement errors (Forbes et al., 2021). AtlasViewerGUI software was used to project the points onto age-specific atlases. An atlas available as part of the software was used for the adults (Aasted et al., 2015) and a 3-year-old head atlas from the Neurodevelopmental MRI database (Richards et al., 2016;Richards & Xie, 2015) was used for the children. Monte Carlo simulations with 100 million photons were run to create sensitivity profiles for each channel and for each adult and child (Fang & Boas, 2009). Image reconstruction techniques were implemented in NeuroDOT to integrate pre-processed optical density time-series data with the subjects’ light sensitivity profiles. This generated voxel-wise time-series data for each chromophore and child/adult (Eggebrecht & Culver, 2019;Forbes et al., 2021). Physiological noise present in hemodynamic data tends to manifest as a global, rather than a local, component (Kirilina et al., 2012) and removing a global component from the dataset has been shown to improve temporal and spatial accuracy in localizing task-relevant changes in the hemodynamic response (Zhang et al., 2016). A global signal regression was run using the NeuroDOT regcorr function, which removed the mean value from the data and therefore removed those changes that were relatively spatially invariant and most likely to be driven by background physiological processes.

Finally, a general linear model (GLM) with a regressor assigned to each learning outcome (words learned, words not learned) was separately run for each chromophore and each adult and child. Each trial was modelled with a 1 s boxcar that was convolved with a hemodynamic response function (HRF) derived specifically from optical imaging (Hassanpour et al., 2014). These analyses generated beta coefficient images for the independent variable, chromophore, and participant. The participants’ resulting beta maps by learning and chromophore were transformed into the MNI atlas space. The participant beta maps in the atlas space were converted into a binary mask for each subject and then were summed across individuals to create a group mask for children and for caregivers that contained voxels with at least 70% of the data points present (missing no more than 30% of the data points). These masks, in relation to the NIRS geometry, are shown inFigure 3.

**Fig. 3.**
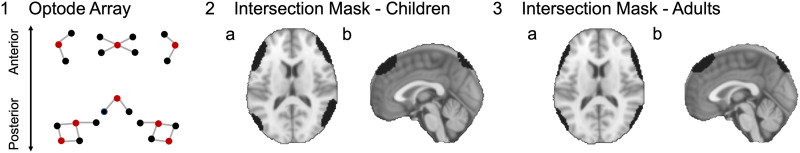
The fNIRS Recording. Note. (1) The geometric array of the NIRS cap, with the front of the head positioned toward the top. Six clusters are arranged bilaterally covering superior/pre/inferior frontal, posterior temporal, and parietal areas of cortical surface. Sources are shown in red, detectors are shown in black, and channels are indicated with grey lines. (2) The final recorded area in dark grey against the brain atlas for children, containing voxels with sensitivity above the threshold for 70% of participants, shown from (a) the top and (b) the side and (3) The corresponding mask for adults shown from the (a) top and (b) side.

### 2.9 Statistical analyses

A logistic regression statistic and a Pearson correlation coefficient were first calculated to determine whether adults and children learned words independently and at different rates, respectively. Then, a t-test compared the mean number of words learned by age. Pearson correlation coefficients related the child’s composite vocabulary score, and the rates of naming from adults and children, to the number of words children learned.

The canonical hemodynamic response to a stimulus has been modelled in past work as going in opposite directions for changes in the concentration of each chromophore (Pinti et al., 2020), and this form has also been assumed in the current pipeline (Forbes et al., 2021). Therefore, we limited the neuroimaging analysis to those clusters that showed a statistical interaction between theΔHbO_2_and theΔHbR concentrations. Both models were run using the 3dLME function in the Analysis of Functional NeuroImages (AFNI) program, as outlined directly below:

Children’s data: lmer(child’s age group (32 months vs. 54 months) * child’s learning outcome (learned vs. not learned) * chromophore (HbO_2_vs. HbR) + (1|participant))
Adults’ data: lmer(child’s age group (32 months vs. 54 months) * child’s learning outcome (learned vs. not learned) * chromophore (HbO_2_vs. HbR) + (1|participant))

In each model, the child’s age (32-month group versus 54-month group), learning (whether the word was learned or not), and the chromophore (HbO_2_versus HbR) were included as fixed effects, while the participant ID was included as a random effect. The effects of interest considered in these models were the main effect of age, main effect of learning, and the interaction effect between age and learning.

In each case, spatial autocorrelation within the model residuals was examined afterwards to estimate an appropriate cluster size to eliminate family-wise error at alpha = .05, using the 3dClustSim function in AFNI (Cox et al., 2017). The images were then thresholded at this cluster size with a voxel-wise*p*-value of less than .01 for the corresponding F-statistic, using 3dClusterize. The minimum cluster size was 149 voxels for children and 77 voxels for adults. The voxel dimensions were 2 mm x 2 mm x 2 mm. Averaged HbO_2_and HbR concentrations were extracted from significant clusters using 3dROIstats for display and further analyses. The extracted clusters were all significant atp<.01. An average for each age, learning and chromophore was extracted from each cluster for each participant.

We conducted equivalent linear models in R: lm(age group (32 months vs. 54 months) * learning outcome (words learned vs. words not learned) * chromophore (HbO_2_vs. HbR)). Prior to running these models, boxplots were created for each learning outcome and chromophore and outlying values were replaced with the remaining sample mean, using the boxplot.stats function to identify values farther from the interquartile range than± 1.5times its length. We removed missing data (values of 0) before replacing the outlying values, as reported inTable S1. Standardised beta coefficients and test statistics are reported from the model outputs. Post hoc comparisons were finally run on the significant interactions via Welch-adjusted t-tests, and the resulting contrasts were compared with the appropriate Bonferroni-adjusted threshold for statistical significance.

## 3 Results

### 3.1 Behavioural metrics

The mean BPVS-3 (composite vocabulary) score for 32-month-olds was 37.26 (SD=12.99), and 69.59 for 54-month-olds (SD=9.95). As shown inFigure 4, children’s learning over the entire experiment ranged from 1-8, out of 8 possible, total object names. On average, 32-month-old children learned 4.0 words during the study (SD=2.0), while 54-month-old children learned 5.4 words (SD=2.0). Bonferroni-corrected pairwise contrasts confirmed that children learned the names of the objects with equivalent likelihood (for all$\chi^{2}\left(1\right),$p>.05). Because the object set size did not predict whether children were more likely to learn a given word,z=1.45,p>.05the analysis combined the set sizes. Adults and children learned different object names,z=1.43,p=.152, and the number of words adults learned was not correlated with the number of words their child learned,r(35)=.057,p=.736.

**Fig. 4.**
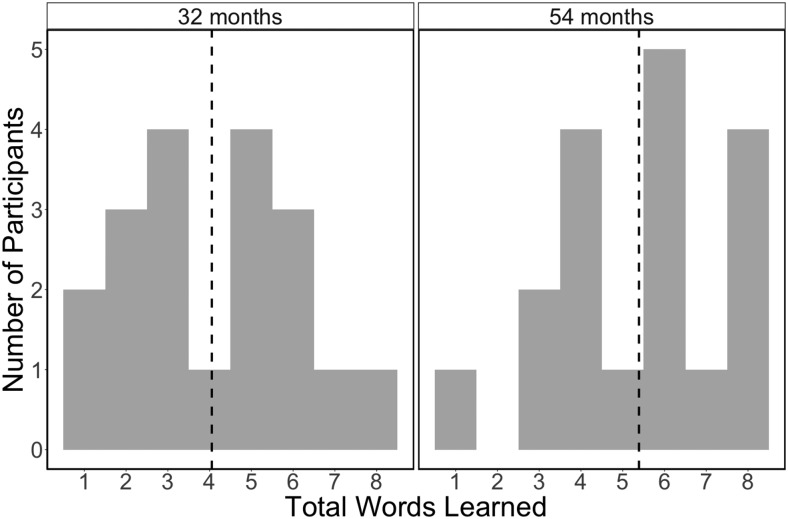
Overall Rates of Children’s Learning by Age Group. Note. Histograms that show the total number of words learned on the x axis, and the number of younger (left) and older (right) children who learned that number of words. The mean number of words learned for each age group is indicated with a vertical, dashed lined, showing a qualitative, but non-significant, difference.

Adults named each object an average of 12.61 times, (SD=4.76). Children named each object far less, at an average of 4.45 times with a proportionally higher variability (SD=3.41). The mean rate of adults’ object naming was not correlated with the number of labels children learned,r(35)=.104,p=.540, nor was the mean rate of children’s naming correlated with the words they learnedr(35)=.164,p=.331. By contrast, overall vocabulary scores were strongly correlated with how many words children learnedr(34)=.464,p=.004, and as expected, older children learned more words than younger children, though this was only a marginally significant difference,t(35)=1.99,p=.054(seeFig. 5).

**Fig. 5.**
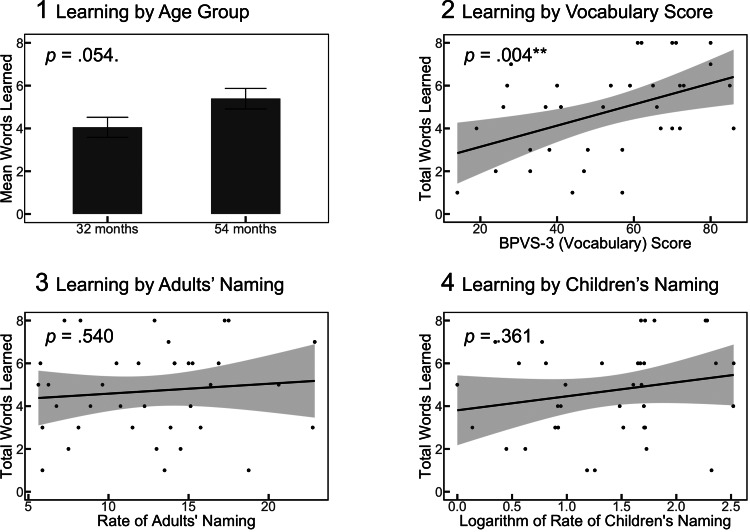
Relationships Between Children’s Learning and Age Group, Vocabulary, and the Frequency of Object Naming. Note. Relationships between children’s learning and age, vocabulary and rates of naming. (1) A marginally significant relationship was observed between the mean number of words learned by the 32-month-old children and the mean number of words learned by the 54-month-old children. (2) Composite BPVS-3 (vocabulary) scores were strongly correlated with the number of words children learned during the experiment, but (3-4) no association was observed between how many times the adult or child named the objects and word learning. This suggests that the quality, rather than the quantity, of the naming events would be a better predictor of learning during the interactions, as explored in the subsequent neuroimaging analysis. ·p<.10, **p<.01.

In summary, the behavioural results showed a robust expected positive relationship between vocabulary scores and word learning in the task, and a weak difference in word learning between the age groups in the expected direction (with older children learning slightly more). The control measures also alleviated specific concerns about confounding variables in the study. Learning did not differ as a result of the amount of times an object was named. Therefore, the neural findings would not confound the relative frequency of hearing an unfamiliar label with neural signatures that differentiated learned naming events from those not learned. Because the adults and children learned different object names, the adults’ event-related responses to words their child learned versus those their child did not learn could be considered independently from their own learning during the same interactions.

### 3.2 fNIRS analysis

Median-centred Levene tests that contrasted the learned versus not learned conditions revealed that these variances did not differ for the children:F(1,120)=3.14,p=.079, or for the adults,F(1,124)=1.30,p=.255. Further, the absolute value of the amplitudes did not differ by learning for the children, Welch-adjustedt(112)=1.79,p=.075, or for the adults, Welch-adjustedt(100)=−1.11,p=.269. Therefore, the learning conditions did not differ in their absolute magnitudes of amplitudes nor show different amounts of variance. Within the beta averages by condition and chromophore for those clusters found in the main models, the results from the final linear model are reported inTable 1below and are shown inFigures 6and7.

**Table 1.** Standardised regression coefficients from the models applied to fNIRS data.

	Vol. centre								
Effect	Region	Vol.	Hemi	x	y	z	β	t	p
Children									
Hb	SmG	203	R	54	-49	46	.31	3.68	< .001
LearnedxHb	pSTC	287	R	58	-56	12	-.38	-4.70	< .001
Adults									
LearnedxHb	AG	165	L	-54	-68	16	-.14	-1.67	.098

Note. A summary of the effects found in the main models run for the children and for the adults, corrected for outlying values. Only those effects that showed a statistical interaction with chromophore (HbO vs. HbR) were considered in this analysis.

**Fig. 6.**
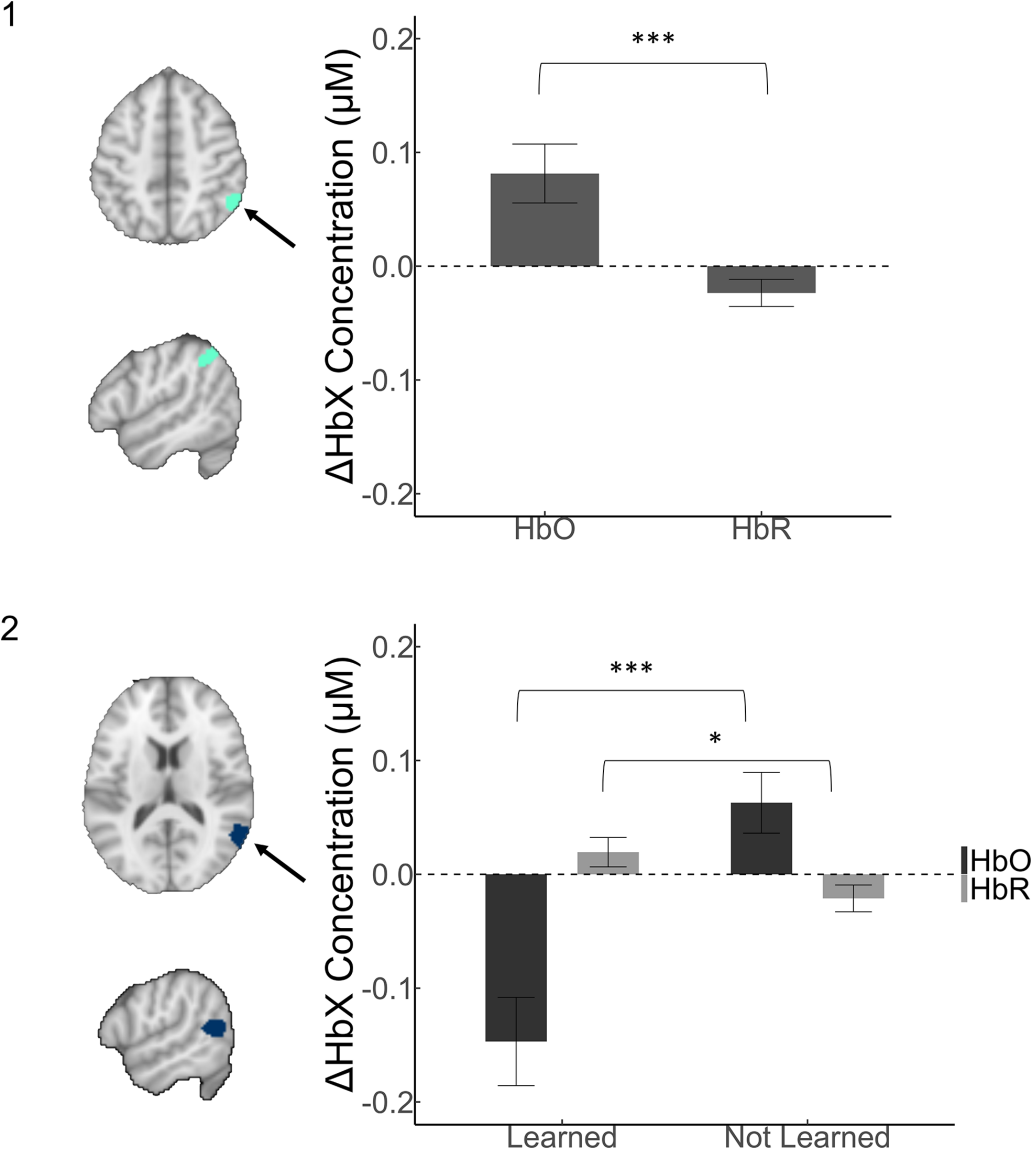
Visualisations of Children’s Hemodynamic Responses. Note. The significant within-cluster beta values expressing the relative changes in the HbX concentration for children. All effects are shown in (upper) an overhead orientation and (lower) a side orientation. The arrow points to the activated region. Navy blue regions show negative-going effects and pale green regions show positive-going effects. (1) Whenever children heard the name of a new object spoken, this positively activated the right supramarginal gyrus (within the IPL) and (2) An interaction between learning and chromophore was further uncovered in the right posterior temporal cortex. This region was relatively inactive or suppressed (depending on interpretation) during naming events where children were learning and generally positively activated when they were not. To evaluate the strength of specific contrasts, t-tests were run with a Welch’s adjustment applied and compared to the significance level required with a Bonferroni adjustment. Within these contrasts: *refers top<.05; **refers top<.01; ***refers top<.001.

**Fig. 7.**
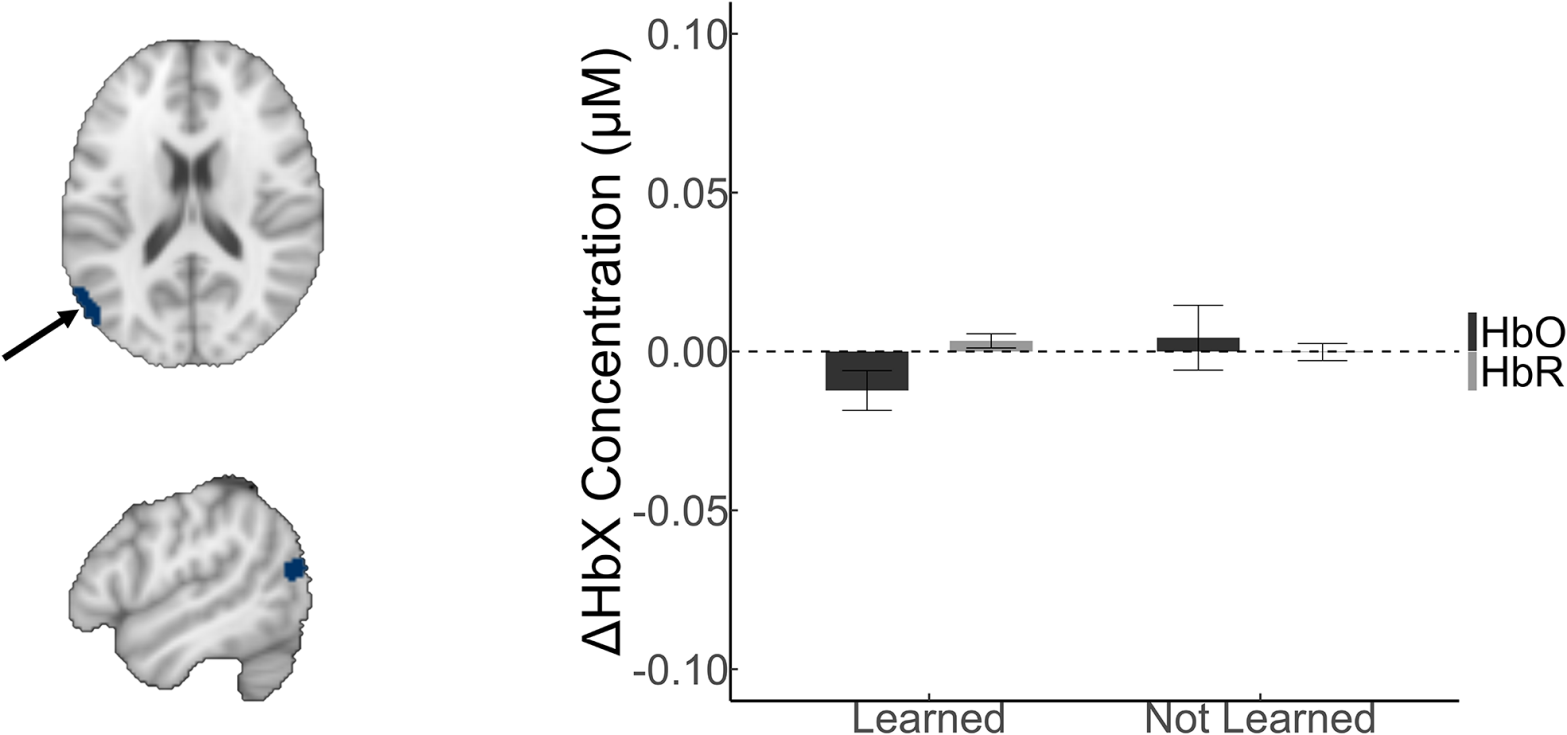
Visualisations of Adults’ Hemodynamic Responses. Note. The non-significant within-cluster beta values expressing the relative changes in the HbX concentration for adults near the left angular gyrus. The arrow points to the activated region.

As shown inFigure 6and reported inTable 1, a main effect of chromophore revealed a common response across all of the instances of unfamiliar object labelling in the supramarginal gyrus. Positive activation in this area was observed in the seconds following hearing a new object labelled in the middle of dialogue, isolating this activation from activation that occurred more generally as the caregivers and children conversed. In addition, an interaction between learning and chromophore was observed in the right posterior temporal cortex. As reported below inTable 2and shown above inFigure 6, greater positive activation for changes in HbO_2_concentration, and greater amplitude negative-going activation for HbR concentration characterised the neural responses when children did not learn the names of unfamiliar objects. By contrast, when they did learn, the neural responses were characterized by reduced amplitudes.

**Table 2.** Post-hoc contrasts.

	Effect	Contrast	Hb	t	df	p
Children	LearnedxHb	Learned-not learned	HbO	-4.46	59	< .001
	LearnedxHb	Learned-not learned	HbR	2.33	63	.023

In summary, the results uncovered event-related responses to instances of naming for the children during learning that were outside of left-lateralised language-predominate areas in the adult literature. The hemodynamic response was lesser in amplitude as children were learning and exceeded the expected amplitude when they were not, as shown inFigure 6. By contrast, the study did not produce evidence that adults’ hemodynamic responses differed when presenting word-object pairings that their child learned, compared with those they did not learn. The region uncovered in the initial analysis turned out to be dependent on a few influential values, as summarized inTable S1, and was not significant anymore after correcting for outlying values, as shown inFigure 7.

## 4 Discussion

What information do teachers and learners actively process during interactive word learning? This analysis most notably uncovered evidence of an effect of learning at the neural level for children that was time-locked to the adults’ labelling of referents, through classifying and comparing event-related averaged responses during these naming events based on children’s learning outcomes. An additional, general effect during the naming events was also uncovered (a main effect of chromophore). By contrast, the analysis did not uncover straightforward evidence of differences in language processing between words learned and not learned for children and left parallel signatures for adults needing further work to fully clarify. To interpret these results further, we took two different approaches. First, we considered a handful of closely related studies to determine the extent to which past work might align with the hypothesis of the study. Second, we conducted exploratory searches within the Neurosynth meta-analytic software^1^to determine the functional associations in past work with the centre of mass of the regions activated in the study, weighted by the number of studies that had found activation within the same locations.

## 4.1 Responses to naming events

The regression models revealed differences within each chromophore between moments of learning and not learning in the children’s hemodynamic responses to naming events, independently of their overall rates of learning, as well as a main effect of chromophore (an effect that was common across the naming events). The study findings were not within well-established pathways for language processing as expected (Lipkin et al., 2022;López-Barroso et al., 2013;Romeo, Leonard, et al., 2018;Romeo, Segaran, et al., 2018, also seeHirsch et al., 2018;Stephens et al., 2010). However, the study hypothesis considered the possibility that in this younger cohort, rightward activation could be observed. Thus, it seems possible that the children’s results could have mapped onto broader regions still correlated generally with lexical processing during conversation (Gow Jr, 2012), narrative (Xu et al., 2005), and single word presentation (Davis, 2016), if lateralisation is factored out based on the knowledge that language processing increases in leftward lateralisation with age. Given that these were responses specifically time-locked to hearing object names, it seems plausible that the activation observed would directly support the formation of corresponding word-object associations (De Benedictis et al., 2014).

Alternatively, when referenced to predominately adult work and in an exploratory manner, the full model uncovered correlates that were functionally associated with social and task-relevant processing, as described below^1^, with the returned associations reported inTable S2. For children, an interaction between learning outcome and chromophore occurred in the right posterior temporal cortex. Functionally, this area was associated via the Neurosynth database with interpreting motion and other dynamic social input and with the temporoparietal junction (TPJ), suggesting that the effect may relate to the integration of social information with the naming event. A main effect of chromophore in the nearby right supramarginal gyrus, within the inferior parietal cortex, potentially revealed that children showed briefly increased task-driven attention when hearing an object named, also according to the results from the database search. This effect that was specific to the unfamiliar object labels seems to be consistent with that of a large-scale study of word processing in older children, where greater posterior right-hemisphere activation, like that observed in this study, was observed for lower-frequency words (Sugiura et al., 2011). In this case, the lower frequency, or more surprising, words would be the unfamiliar object names that were spoken during regular conversation.

Our findings in the right hemisphere may be explained by two differences in approach from previous work. First, we report an analysis that covered the full recorded area, rather than constraining that analysis to test for differences within the left-lateralised language network specifically. So, it is possible that smaller regions may have been activated in the left hemisphere or that the differences could have had a lesser amplitude, than we detected here. Second, coverage of the temporal cortex did not overlap as clearly with the language network (owing to the cap construction and proximity to the ears) as the inferior frontal cortex, so it was consequently not as clear whether we had the appropriate coverage and power to detect differences in relevant parts of the temporal cortex involved in speech comprehension. Nevertheless, the inferior frontal gyrus is indicative of language comprehension during dialogue (Arvidsson et al., 2024), suggesting that if activity had occurred in Wernicke’s area, for example, we would likely have observed some difference in Broca’s area, too. Third, the effects we observed occurred over the course of a brief learning episode, whereas in past work, language experience had presumably driven changes in the plasticity of the language network over time (Romeo, Segaran, et al., 2018) or induced plasticity in regions subserving attention over a period of weeks of learning new words (Ekerdt et al., 2020). To further support this, right-lateralised correlates of language processing have been more closely associated with narrative (Xu et al., 2005) and with more naturalistic speech (Alexandrou et al., 2017) than with isolated single word or sentence processing. These results suggest that neural signatures of learning that are time-locked to instances of naming during an interaction should not necessarily only involve hypotheses about the language network as defined in such work, but may really reflect contextual differences and processes that support learning.

The direction of the main finding with children, that, is, lesser activation during learning as opposed to during and just after the naming of words that were not learned, seems to indicate that learning occurred during straightforward naming events with fewer demands to resolve situational ambiguity. In other words, this effect may reveal that markers of word learning occur during straightforward naming events when it is easier to understand the intended label-referent pairing and there is less need to make additional effort to interpret social information. The pattern of results found here is generally consistent with those of other studies that have tested for differences in the neural correlates of sentence processing in contexts that were relevant to social interaction in adults (Egorova et al., 2016;Redcay et al., 2016;Rice & Redcay, 2016) and children (Rice et al., 2016). However, the present results may be the first to find relatively less activity during learning from instances of naming in the posterior temporal cortex. For example, it may be the case that children’s reasoning about the speaker’s intentions and/or actions at the point of naming drove the learning effect, though notably, greater activation during the naming of words not learned may reveal that this type of effort is a better indication of what is going wrong when learning is not occurring, an explanation that is also potentially consistent with the clear distinction between semantic and action-related processing of object labels observed previously (Egorova et al., 2016). The finding is also consistent with recent work revealing that functional connectivity in the bilateral posterior superior temporal cortex may be robustly related to social communication skills in toddlers, but not to language development specifically (Smith et al., 2023).

Finally, the reduced activity observed during learning is consistent with learning effects observed in other studies. For example, after being trained in multiplication problems, children showed decreased activation in the parietal cortex within fNIRS recordings, while shifts in strategy that had been reflected in the neural data from adults were not observed (Soltanlou et al., 2018). There, a signature of learning that was reflected by reduced amplitude was observed in children without the power to detect more subtle changes in activation that are part of the overall learning signature as observed in adults. This seems to also fit indirectly with the effect of spoken word repetition as reduction in brain activity in infancy (Emberson et al., 2017) and potentially also with increased activity in the right temporal cortex after hearing an unpredictable stimulus in adulthood (Muñoz-Caracuel et al., 2024). In summary, the present results seem to best align with the reduced responses observed as a signature of predictability and learning more generally, across a wider variety of paradigms. Meanwhile, associations in the literature that are specific to the region where differences by learning were uncovered suggest that greater effort to resolve social and other contextual ambiguity during object naming occurred when word learning was not taking place.

## 4.2 Limitations and future directions

The study generated some questions that may be tested in future designs. The first, most evident need is for larger experiments with greater statistical power to (a) be able to further substantiate the findings with children that are strongly indicative in this design and (b) determine whether differences exist in adults’ hemodynamic responses to naming events that vary, specifically, based on whether their child is learning. The design presented here could be applied to larger studies in the future to provide greater support for these results and to resolve the inconclusive findings, most notably, that with the adults. The second is to understand why, through measuring and factoring in looking, dialogue content or turn-taking during the interactions that could provide a basis for examining mutual prediction (Hamilton, 2021), variables that we were not able to consider, here.

Some limitations were present in the behavioural part of the design. In this case, some objects had vaguely familiar characteristics, as can be seen inFigure 1and again inFigure S1. In the future, objects could be designed and 3D printed in order to guarantee that their shapes are completely unfamiliar. Additionally, the child’s learning was assessed several minutes after being taught the unfamiliar objects’ names, and this timeframe deserves critical consideration both in relation to word learning, and to the moment-to-moment referent selection that the word learning was related to in the fNIRS analysis (Horst et al., 2006). Findings from past studies that included delays of 5 min indicate that this gap would have allowed some consolidation in long-term memory to occur between the learning interactions and the test (Horst & Samuelson, 2008). However, these processes (fast mapping and word learning) have been shown to be related continuously (Kucker et al., 2015) and a test given after the day of the experiment would provide a clearer measure of long term lexical retention (Vlach & Sandhofer, 2012). The test also did not require children to produce or spontaneously use the new labels in conversation after having learned them during the interaction, only to consistently select the named referents.

Though this may have been the first study to compare event-related responses to object naming based on learning during extended openly structured interactions, the results did not support that local changes within caregivers’ functional brain activation will reliably show some kind of evidence of successful or unsuccessful teaching. However, what determines the relationship between adults’ brain activity when teaching their child, and their child’s learning, must reflect the interaction they are having at linguistic and behavioural levels. Recent work has found some evidence that interpersonal neural measures, such as indices of successful teaching, vary and likely do so with the ongoing dynamics of these interactions. These include the temporal and behavioural sensitivities that drove inter-brain relationships in recent related work (Nguyen et al., 2021,^2023^;Piazza et al., 2021). While there was no evidence in the present study that caregivers responded differently when their child was learning from their naming of objects compared with when they were not, a subset of influential values in the distribution were robust enough in the parietal cortex to identify this region within the full statistical map. Therefore, one possible path forward for future studies is to develop and apply automated approaches for examining dialogue content around these events and throughout the interactions in order to be able to analyse what leads up to and causes individual differences in neural indications of successful/unsuccessful teaching. Because the learning effect for children was uncovered in an area associated with interpreting dynamic social stimuli in previous work, future studies may also consider monitoring moment-to-moment motion and looking to see if these behaviours during instances of naming for learned words are more predictable than those during words that are not learned.

Given the naturalistic testing paradigm, we could not control the temporal proximity of the naming events, which are shown inFigure 2. For this study, the distribution of median spacing between naming events showed that many of these intervals were briefer than the average estimated peak of the hemodynamic response, with a median of just slightly more than 5 sec (Hassanpour et al., 2014). Nor was it possible to control for background factors that may have influenced observations of event-related hemodynamic responses to instances of naming, as these results could have reflected a broader window including seconds immediately around the naming events. However, past fNIRS work has rarely reported event-related hemodynamic responses to object labelling in a similar task to compare. In one closer MRI study, the neural responses to a choice involving concrete nouns (pancakes or fruit for breakfast) that most closely aligned with our results reflected differences originating outside of the choice window where the naming of referents occurred, suggesting that our finding, too, may have reflected differences in social processing effort that consistently occurred*around*the time of the naming events (Rice et al., 2016). Additionally, there is always a chance that task-related differences observed in NIRS results can be confounded by physiology, like heart rate and breathing. We have taken steps to mitigate this through only considering clusters that strongly indicated a canonical hemodynamic response to event-related stimuli and not to any other factor (i.e., those clusters that showed an interaction with chromophore), but this remains another limitation of the study.

Finally, this study did not find evidence of age differences. A marginally significant trend was observed between learning outcomes, and no differences were found in the neuroimaging analysis. The neuroimaging results of this study revealed commonalities between the age groups, yet the behavioural contrast also showed a trend toward an overall difference in learning capacity between the age groups that was not strong enough to be significant. Larger, higher-powered studies may illuminate whether smaller or more continuous differences exist in the neural correlates of learning between these ages. This open question presents a clear avenue for future work: to determine what changes about language learning during interactions between 2 and 5 years of age at a neural level. This question seems to point again to the potential noise levels present in a naturalistic word learning experiment, both in terms of measuring learning, and in terms of measuring neural responses during learning, and the need for larger studies with an improved effect-to-noise ratio in order to observe any more subtle developmental changes.

## 4.3 Concluding points

In summary, the study addressed a gap in previous work of mapping the neural correlates of interactive learning through investigating adults’ and children’s cortical responses to instances of the adults’ naming of unfamiliar objects. The results indicated that for children, moments of learning may be easier, ultimately eliciting less robust neural effort to resolve ambiguity and more straightforward encoding, when compared with moments that did not solidify into accurate word-object mappings. Future work may provide more information about adults’ hemodynamic responses during successful and unsuccessful teaching and the behavioural factors that modulate event-related neural data during interactions.

## Supplementary Material

Supplementary Material

## Data Availability

The code required to run the analyses, with minor adaptation, is available athttps://github.com/developmentaldynamicslab/MRI-NIRS_Pipeline. The code for digitizeR is available athttps://github.com/samhforbes/digitizeR. Ethical approval for the study did not include permission to publicly share the fNIRS data; therefore, the raw data can be made available upon a reasonable request to the corresponding author and that includes institutional ethical approval.

## Footnotes

1 Yarkoni et al. (2011); searches in Neurosynth were made in 2023-2024.
