# Supplementary material for: Neural signatures of word learning during adult-child interactions
Source: Imaging Neurosci (Camb). 2025 Jan 2;3:imag_a_00407. doi: 10.1162/imag_a_00407 (PMC12319735; doi:10.1162/imag_a_00407)
Supplement: Supplementary Material [file imag_a_00407-supp.pdf]

Supplemental File

Testing Materials

The unfamiliar words used in the study were randomly selected from the Novel Noun and Unusual Object Database (Horst & Hout, 2016). They were: lep, tanzer, deld, agen, spoov, reda, yok and modi. These names were counterbalanced across the sessions such that each novel noun was assigned to each object with an approximately equal frequency.

Figure S1

Photos of the Objects

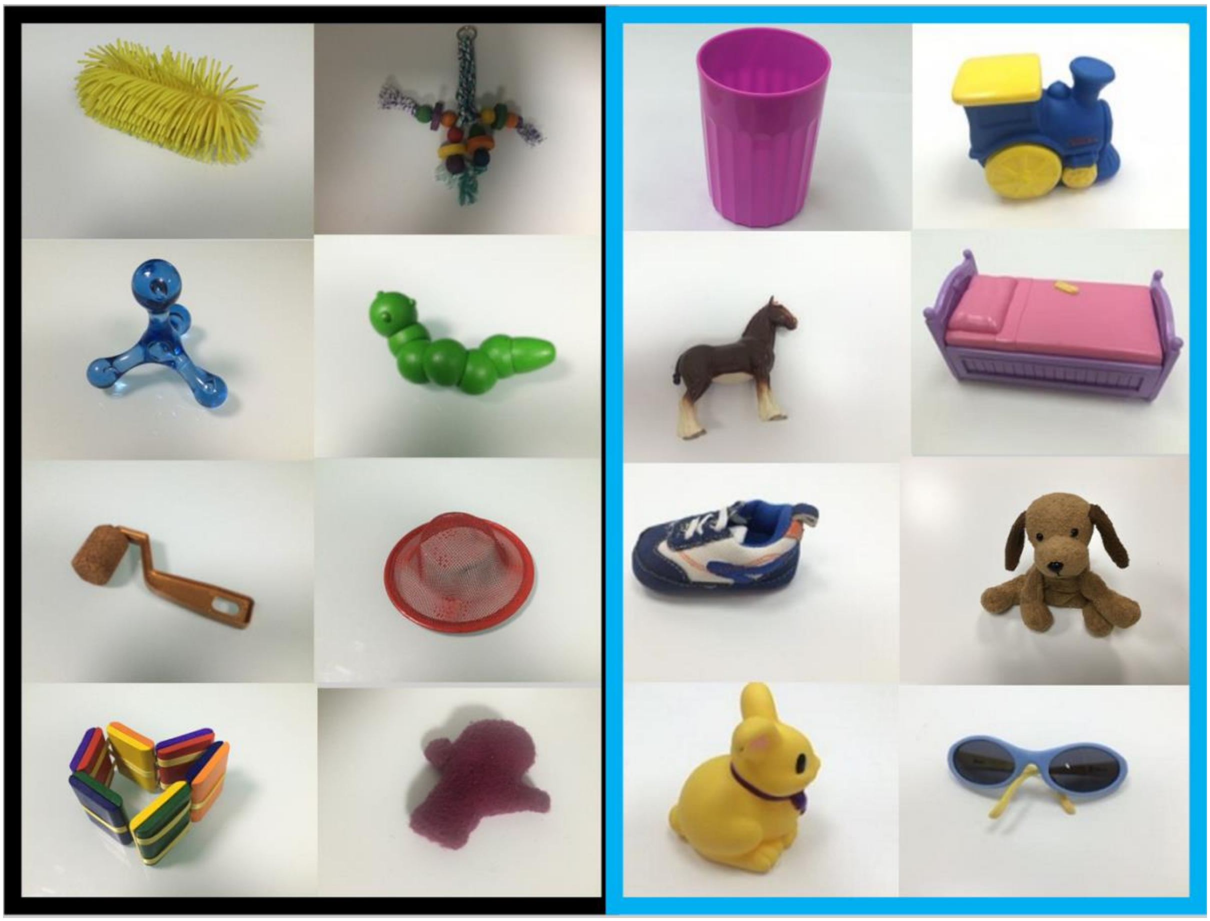

Note. The unfamiliar objects on the left are framed by a black rectangle, and the familiar objects on the right are framed by a blue rectangle.

fNIRS Cap Geometry

Figure S2

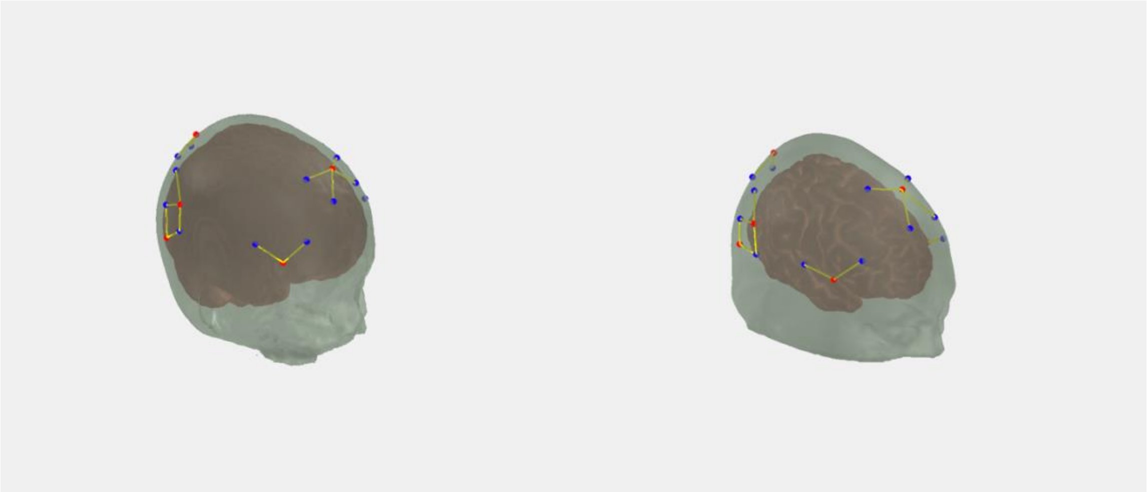

Note. The positioning of the cap geometry for a 4-year-old child (left) and an adult (right), that used a 3-year-old head atlas for the child's data (Richards et al., 2016; Richards & Xie, 2015) and the atlas that was part of the Homer2 processing package for the adult data (Aasted et al., 2015).

Table S1

Summary of Missing and Replaced Data in the Study

	Chromophore	Exp. Cases	Values of 0	Outliers
Children-Hb				
32 mo - L	HbO_2	19	3	
	HbR	19	3	1
32 mo - NL	HbO_2	18	2	1
	HbR	18	2	
54 mo - L	HbO_2	18	1	
	HbR	18	1	1
54 mo - NL	HbO_2	14	1	1
	HbR	14	1	
Children-LearnedxHb				
32 mo - L	HbO_2	19		
	HbR	19		2
32 mo - NL	HbO_2	18		3
	HbR	18		3
54 mo - L	HbO_2	18	2	1
	HbR	18	2	2
54 mo - NL	HbO_2	14	2	2
	HbR	14	2	2
Adults-LearnedxHb				
32 mo - L	HbO_2	19	3	5
	HbR	19	3	4
32 mo - NL	HbO_2	18	3	3
	HbR	18	3	1
54 mo - L	HbO_2	18		2
	HbR	18		2
54 mo - NL	HbO_2	14		2
	HbR	14		3

L = Learned, NL = Not Learned

Note. There were fewer beta maps for the words not learned, as shown in the expected cases column, because five children in the study, one 32-month-old and four 54-month-olds, learned all eight words.

Table S2

Keywords Returned by the Neurosynth Cluster for Significant Results

Effect	x	y	z	association
**Children**				
Hb[1]	54	-48	46	parietal
				inferior parietal
				response inhibition
				cortex inferior
				inhibition
				frontal parietal
				frontoparietal network
				monitoring
				cognitive control
				execution
LearnedxHb[2]	58	-56	12	motion
				temporal sulcus
				social
				temporo
				psts/sts
				sulcus
				temporo parietal
				junction
				parietal junction
				posterior superior
				face
				social interactions
				intentions
				superior temporal
				social cognitive
				gaze
				vision
				actions
				temporal
				posterior
				person
				mind tom
				theory mind
				mental state
				tom
				social cognition
				faces
				visuo
				object
				listened
				viewing

Note. The exact location and associations with Z-scores > 3.0 for the regions uncovered in the study, as returned by neurosynth.org.

Supplemental References

Aasted, C. M., Yücel, M. A., Cooper, R. J., Dubb, J., Tsuzuki, D., Becerra, L., Petkov, M. P., Borsook, D., Dan, I. & Boas, D. A. (2015). Anatomical guidance for functional near-infrared spectroscopy: AtlasViewer tutorial. *Neurophotonics*, *2*(2), 020801:1-16. https://doi.org/10.1117/1.NPh.2.2.020801

Horst, J. S. & Hout, M. C. (2016). The Novel Object and Unusual Name (NOUN) Database: A collection of novel images for use in experimental research. *Behavior Research Methods, 48*(4), 1393-1409. https://doi.org/10.3758/s13428-015-0647-3

Richards, J. E., Sanchez, C., Phillips-Meek, M. & Xie, W. (2016). A database of age-appropriate average MRI templates. *NeuroImage*, *124*, 1254-1259. https://doi.org/10.1016/j.neuroimage.2015.04.055

Richards, J. E. & Xie, W. (2015). Brains for all the ages: Structural neurodevelopment in infants and children from a life-span perspective. In *Advances in Child Development and Behavior* (Vol. 48, pp. 1-52). JAI. https://doi.org/10.1016/bs.acdb.2014.11.001
